# Acoustoelectric Current in Graphene Nanoribbons

**DOI:** 10.1038/s41598-017-01979-8

**Published:** 2017-05-11

**Authors:** T. Poole, G. R. Nash

**Affiliations:** College of Engineering, Mathematics and Physical Sciences, University of Exeter, EX4 4QF Exeter, UK

## Abstract

Surface acoustic waves (SAWs) propagating on piezoelectric substrates offer a convenient, contactless approach to probing the electronic properties of low-dimensional charge carrier systems such as graphene nanoribbons (GNRs). SAWs can also be used to transport and manipulate charge for applications such as metrology and quantum information. In this work, we investigate the acoustoelectric effect in GNRs, and show that an acoustoelectric current can be generated in GNRs with physical widths as small as 200 nm at room temperature. The positive current in the direction of the SAWs, which corresponds to the transportation of holes, exhibits a linear dependence on SAW intensity and frequency. This is consistent with the description of the interaction between the charge carriers in the GNRs and the piezoelectric fields associated with the SAWs being described by a relatively simple classical relaxation model. Somewhat counter-intuitively, as the GNR width is decreased, the measured acoustoelectric current increases. This is thought to be caused by an increase of the carrier mobility due to increased doping arising from damage to the GNR edges.

## Introduction

The transportation of charge in low-dimensional electron systems has attracted significant attention in recent years as a potential route towards nanoscale electronic devices. Surface acoustic waves (SAWs) propagating on piezoelectric materials provide a unique tool for studying and realising such systems. The strong interaction between the piezoelectric fields associated with a SAW, propagating on a piezoelectric substrate, and a charge carrier system is known as the acoustoelectric effect^1–4^ and has previously been used to probe, for example, the electronic properties of quantum dots^5^ and wires^6^. Recent efforts, however, have concentrated on the interactions of SAWs with graphene^7–27^, a two-dimensional (2D) carbon allotrope with outstanding physical properties and numerous potential applications^28–30^.

The ability of piezoelectric potentials to trap and transport charge at the speed of sound enables the generation of acoustoelectric currents. These have been measured in micron-scale graphene monolayers^7, 8^, and we have previously studied the acoustoelectric response of large-area graphene sheets produced by chemical vapour deposition (CVD) transferred to LiNbO_3_ SAW devices at room temperature^9^, low temperature^10^, and under illumination^11^. Theoretical and experimental reports have examined DC-biased mono- and multi-layer graphene sheets for SAW amplification^12, 13^, while others have studied the use of electrolytic solutions^14^, and gases^15^ to control the carrier concentration in graphene films, opening up possibilities for low-power chemical sensors. In contrast, by using an ion gel gate to modulate the conductivity of graphene, Bandhu and Nash^16^ demonstrated that graphene can be used to control the SAW amplitude and velocity shift. Optimization of this approach could lead to a practical, cost-effective voltage-controlled velocity shifter suitable for use in wireless sensor applications^16^.

Theoretical studies predict a further variety of physical phenomena arising from SAW-graphene interactions^17–19^, and several groups have demonstrated carbon monoxide, hydrogen^20^, and moisture^21–24^ detectors based on SAW-graphene systems.

Despite several studies highlighting the potential advantages of incorporating graphene and other 2D materials with SAW devices^31^, there is an absence of research on SAW interactions with graphene nanoribbons (GNRs). It is a well-known disadvantage of graphene that its gapless band structure is not suited to integration with digital electronics, which typically require high on-off ratios^29, 32^. Patterning graphene into GNRs can open a bandgap that varies as a function of ribbon width, with appropriate fabrication methods^32^. Additionally, the electric potential associated with SAWs propagating on piezoelectric materials enables the confinement of charge carriers in nearby electronic systems, that could have applications in metrology and quantum computing^3, 4, 33–35^.

In this manuscript, measurements of an acoustoelectric current generated in GNRs fabricated from monolayer CVD graphene transferred to LiNbO_3_ delay lines. The acoustoelectric current was first studied in GNR arrays consisting of 500 nm-wide ribbons, and the measured linear dependence of acoustoelectric current on SAW intensity and frequency is consistent with the interaction between the SAWs and the graphene charge carriers being described by a relatively simple classical relaxation model. We have also investigated the width dependence of the acoustoelectric current in GNRs, with widths ranging from 200–600 nm, with GNRs of different widths all fabricated on the same SAW delay line. The measured increase in acoustoelectric current as the nanoribbon width decreases is attributed to additional p-doping of the GNR edges, raising the Fermi level in the samples and enabling conduction across grain boundaries and charge puddles arising from the polycrystalline nature of CVD graphene.

## Results

**Figure 1:**
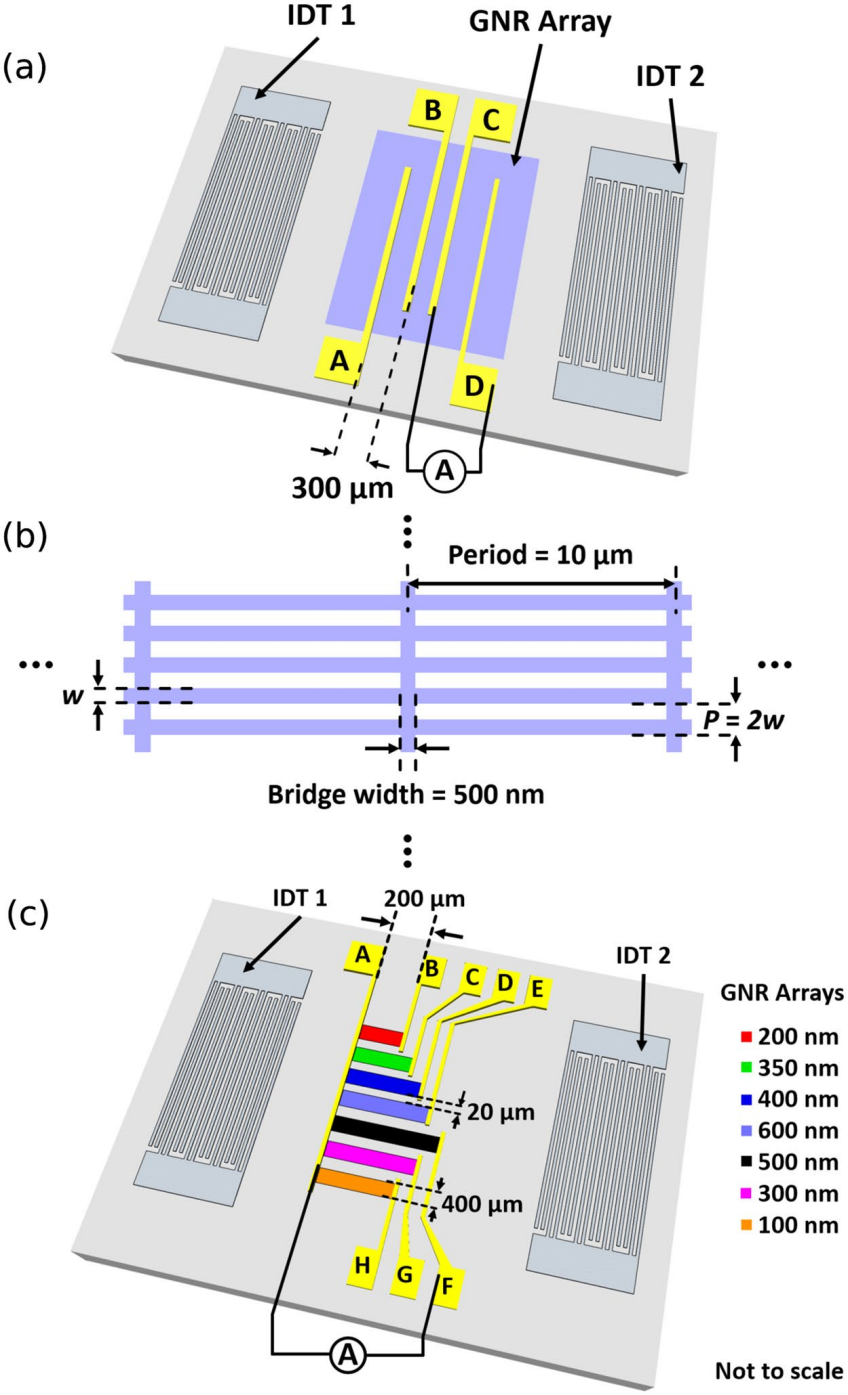
(**a**) A schematic diagram of the device used to study the acoustoelectric current in nanoribbons of a single width. (**b**) A sketch showing the structure of the GNR arrays, including nanoribbon width *w* and period *P*. (**c**) A schematic diagram of the device used to study the width dependence of the acoustoelectric effect in GNRs.

We first consider LiNbO_3_-GNR SAW devices where the nanoribbons are all of 500 nm width. These are arranged in a 2 mm × 3 mm array, shown schematically in Fig. 1(a) (for details of fabrication, see Methodology), where the nanoribbon width is equal to 50% of the ribbon repeat period. The GNRs are connected by a 500 nm-wide perpendicular bridge of graphene every 10 μm to help maintain electrical conductivity (Fig. 1(b)), similar to that used by Luxmoore *et al*. in investigations of GNR arrays on SiO_2_/Si^36^. The use of double-digit interdigital transducers (IDTs) enables the efficient excitation of SAWs at a number of resonant frequencies. The IDTs were pre-defined on the surface by the manufacturer (MESL Microwave/COM DEV International) of the LiNbO_3_ crystal with an acoustic aperture of 3.25 mm and separation distance 5.4 mm, allowing large-area CVD graphene monolayers to be easily positioned between the transducers without shorting them out. The fundamental frequency of 11 MHz (SAW wavelength of 361.7 μm) corresponds to a digit width of 45.2 μm.

**Figure 2:**
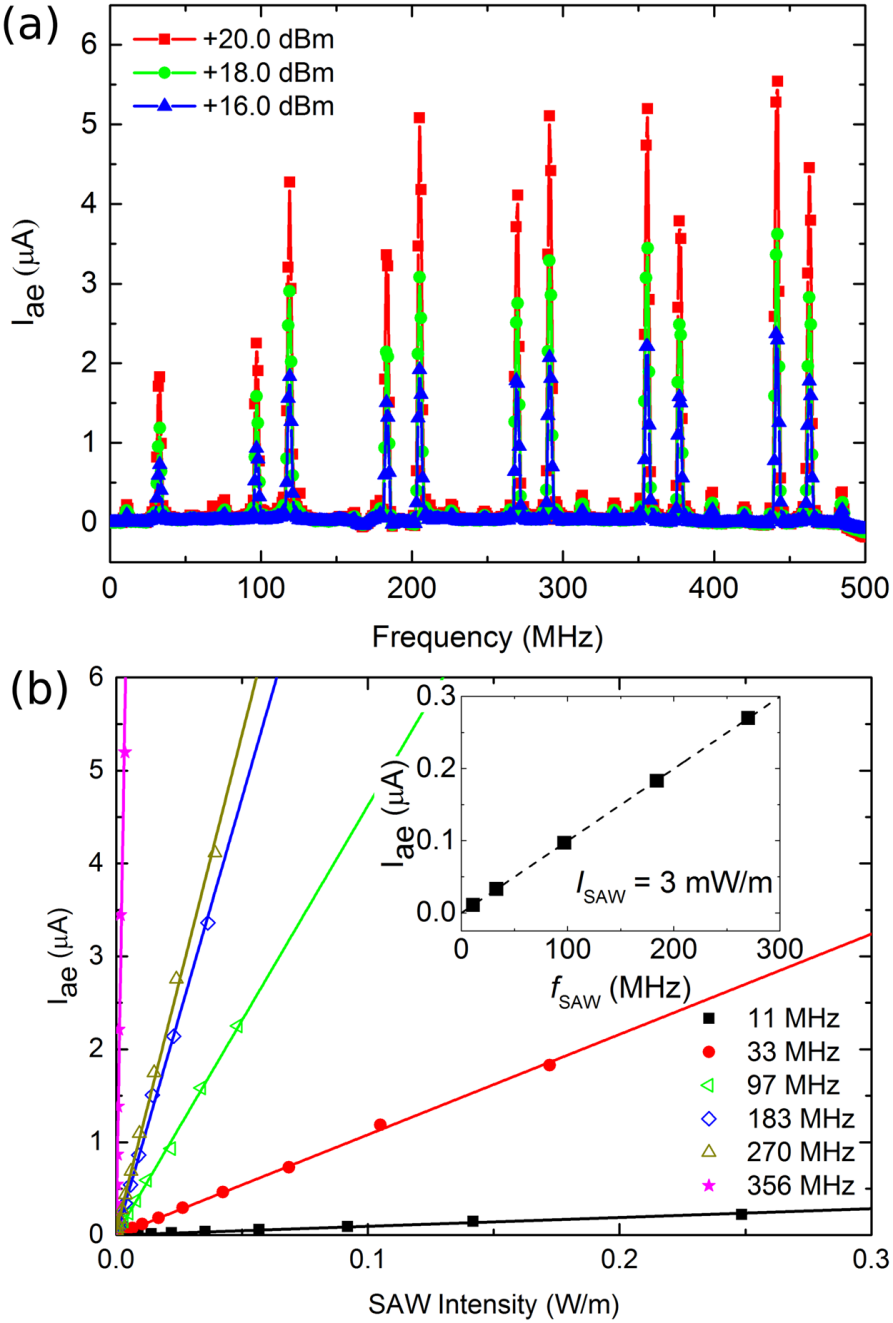
(**a**) Acoustoelectric current as a function of SAW frequency and RF power, as measured between contacts A and B (Device 1). (**b**) Acoustoelectric current as a function of SAW intensity at several SAW frequencies, as measured between contacts A and B (Device 1). Linear fits are guides to the eye. Inset: Acoustoelectric current as a function of SAW frequency for a fixed intensity. I_ae_ is extracted from the linear fits in Fig. 2(b).

Figure 2(a) shows the measured acoustoelectric current (I_ae_) in the GNRs between contacts A and B (separated by 300 μm, with an array width of 3 mm) as a function of SAW frequency and RF power, at room temperature under vacuum. Two devices of this design were studied (Devices 1 and 2), with these results obtained from Device 1. Continuous wave SAWs were excited in increments of 1 MHz in the range 1–500 MHz, and each data point represents the mean of five measurements of I_ae_. The measured current maxima in Fig. 2(a) coincide with the resonances of the IDTs, as observed in our previous work on unpatterned graphene^9^, confirming its acoustoelectric nature. I_ae_ of up to ~5.5 μA was measured for a SAW frequency *f*_SAW_ = 442 MHz and an applied RF power of +20.0 dBm. Acoustoelectric currents of the same order of magnitude were measured between contacts B and C, and C and D, similar to that reported by Okuda *et al*.^14^. This is significantly larger than values we have previously measured on unpatterned graphene samples, but this is due to improvements in the transfer method we use, leading to improved quality graphene film (which we believe results in a higher carrier mobility). This is reflected in room temperature current-voltage measurements of the GNR arrays on Device 1, under vacuum, which yielded values of sheet resistance of: 10.5 kΩ/□ (A to B); 23.7 kΩ/□; (B to C); and 56.8 kΩ/□ (C to D). These values compare to typical values of approximately 100 kΩ/□ we have measured previously^9–11^, including in GNR samples fabricated using our old transfer method The positive sign of I_ae_ in the direction of SAW propagation is indicative of hole transportation, and is consistent with the CVD graphene being p-doped by PMMA residues left during the transfer process^37^. Raman analysis of Device 1 (Supplementary Information) showed the position of G peak at 1588 cm^−1^, a blue-shift also consistent with p-doped graphene^38^. Upon reversal of the SAW direction, the sign of the current also reverses, confirming that the current is induced by the SAWs. Measurements of I_ae_ were found to be stable and reproducible over the course of several weeks. Qualitatively similar results were seen in Device 2, although, due to the significantly higher resistance, 323.6 kΩ/□ between contacts B and C (contact pairs A and B, and C and D were found to be open circuit in this device), the acoustoelectric current was approximately 100 nA for the same SAW frequency and intensity. To rule out possible interference effects, measurements of the acoustoelectric current in Device 2 were repeated with RF frequency increments of 0.5 MHz, but the change in the measured current was negligible.

In Fig. 2(b) the acoustoelectric current is plotted as a function of SAW intensity for several SAW frequencies, as measured between contacts A and B of Device 1. RF power applied to the input IDT ranged from 0 to +20.0 dBm, in increments of +2.0 dBm. To account for the efficiency with which different SAW frequencies are generated, the SAW intensity is estimated from the voltage of the waveform measured at the output IDT, as a function of the input RF power. The relative amplitude of SAWs as a function of SAW frequency in Device 1 is plotted in the Supplementary Information (Figure S1, solid orange curve, square symbols). The range of SAW intensities accessible at each SAW frequency was similar in Devices 1 and 2, and is comparable with those estimated in our previous measurements based on identical substrates^9–11^. A linear dependence of I_ae_ on SAW intensity is confirmed via fitting (solid lines). The inset to Fig. 2(b) shows the linear dependence of I_ae_ on *f*_SAW_ for a fixed intensity of 3 mW/m (dashed line), where the acoustoelectric current at each frequency has been extracted from the linear fits to the measured I_ae_ in Fig. 2(b). These dependencies are consistent with the description of the acoustoelectric effect provided by refs 3 and 4: in the absence of a magnetic field and in a closed circuit, the induced acoustoelectric current density *j* is given by:

1 $$j=-\,\mu Q=-\,\mu \frac{I{\rm{\Gamma }}}{v}$$

where *μ* is the charge carrier mobility, *Q* is the phonon pressure given by *Q* = *I*Γ/*v*, *I* is the SAW intensity, and *v* is the velocity of the SAW (3979 m/s in 128° YX LiNbO_3_^39^). The attenuation per unit length Γ is given by a non-monotonic function of the diagonal component of the conductivity tensor *σ* ^2D^ ^2^:

2 $${\rm{\Gamma }}={K}^{2}\frac{\pi }{\lambda }[\frac{({\sigma }^{2{\rm{D}}}/{\sigma }_{{\rm{M}}})}{1+{({\sigma }^{2{\rm{D}}}/{\sigma }_{{\rm{M}}})}^{2}}]$$

where *K* ^*2*^ is the piezoelectric coupling coefficient (0.056 in 128° YX LiNbO_3_), *λ* is the SAW wavelength, and the attenuation is maximised at a characteristic conductivity *σ*_M_. Bandhu and Nash have reported *σ*_M_ = 10^−7^ Ω^−1^ in graphene-lithium niobate hybrid systems^16^.

In the absence of an electrostatic gate it is not possible to extract a value of the charge carrier mobility in these devices. Wrinkles, rips, tears, and the polycrystalline nature of the CVD graphene are known to limit the mobility^40^, and it is likely that the lithium niobate also impacts the charge carrier mobility. In previous work in similar samples the mobility *μ* was estimated as ~5–10 cm^2^/V.s^9–11^. In Device 1 the relatively high conductivity compared to *σ*_M_ (*σ* ^2D^/*σ*_M_~1000) is expected to yield relatively low acoustoelectric attenuation of SAWs, via Equation (2), suggesting that *μ* in Device 1 is much larger than that previously measured in similar devices^9–11^, consistent with the measurements of resistance. Note also that the SAW attenuation induced by the GNR charge carriers is determined by the conductivity of the graphene nanoribbons on the scale of approximately half of the SAW wavelength^41^. The polycrystalline nature of CVD graphene leads to an inhomogeneous conduction path^42^, which in turn may lead to a SAW frequency dependent mobility and one which is different to those obtained via field effect measurements.

**Figure 3:**
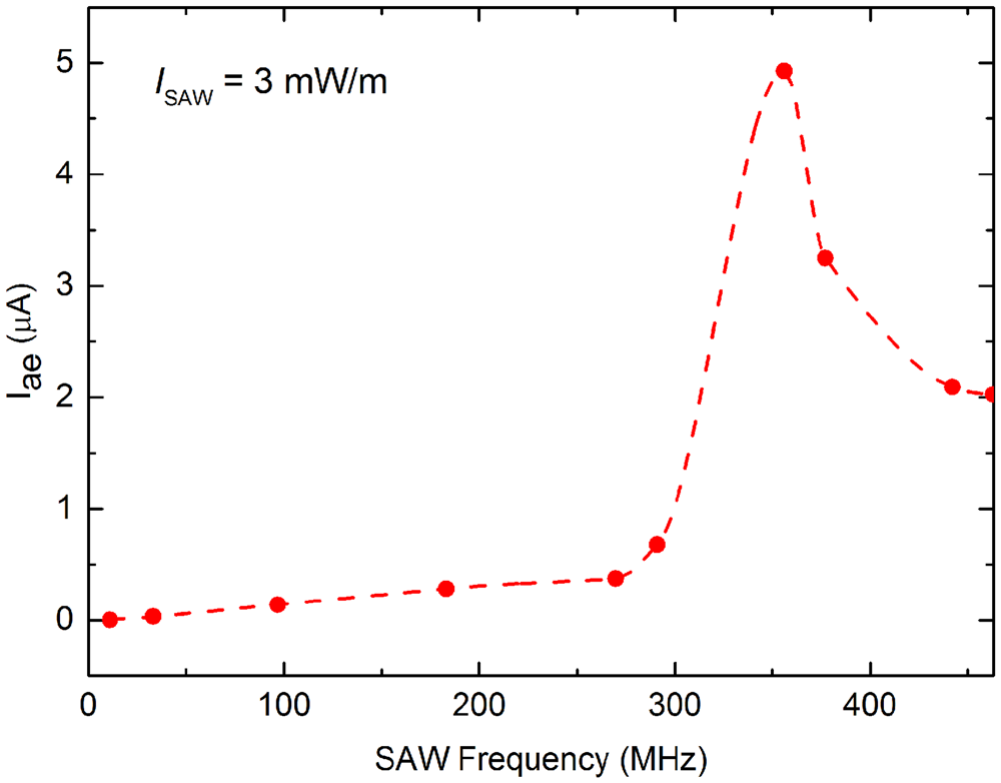
Acoustoelectric current as a function of SAW frequency, extracted from fitting to raw data. Spline (dashed line) is a guide to the eye only. A sharp increase in measured acoustoelectric current is observed at *f*_SAW_ = 356 MHz.

For the frequencies displayed in the inset of Fig. 2(b), I_ae_ shows a strong linear dependence. Beyond this range, we observe a sharp increase in the extracted acoustoelectric current as a function of SAW frequency for a fixed intensity (Fig. 3), which peaks at *f*_SAW_ = 356 MHz. We believe this enhanced piezoelectric interaction between the GNRs and the SAWs at this frequency arises from the commensuration of the SAW wavelength (~11.2 μm) with the periodicity (10 μm) of the perpendicular graphene bridges in the array. This is similar to the recent study by Mayorov *et al*.^25^, in which IDTs were formed from graphene on LiNbO_3_ for the purpose of SAW generation and detection. This coupling could explain the extremely low SAW intensity and relatively large acoustoelectric currents measured for *f*_SAW_ = 356 MHz in Devices 1 and 2. I_ae_ measured between contacts B and C of a third device fabricated without the perpendicular bridge structures did not exhibit such behaviour (see Supplementary Information, Figure S2). This is reflected in Figure S1, where the relative amplitude of SAWs as a function of SAW frequency is plotted for a SAW-GNR device with bridge structures (solid orange curve with square symbols) and without bridge structures (dashed green curve with triangular symbols); the relative SAW amplitude decreases in devices with bridge structures every 10 μm in the GNR array compared to those that do not have bridges, for SAW frequencies of 356–377 MHz (SAW wavelength ~11.2–10.6 μm).

An additional consideration is whether there is a stronger piezoelectric interaction between the SAWs and the graphene charge carriers for SAWs with a wavelength similar to the size of the graphene domains. The graphene used in these measurements has a maximum grain size of approximately 10 μm, according to the manufacturer. The absence of an increase in acoustoelectric current when the SAW wavelength is similar to the domain size (as is the case for *f*_SAW_ = 356 MHz) in the device without bridge structures (Figure S2) suggests that, at room temperature, any effects on I_ae_ of the commensuration of the graphene grain size with the SAW wavelength are negligible.

To investigate the effect of the GNR width on the acoustoelectric interaction, multiple nanoribbon arrays were fabricated on the same LiNbO_3_ SAW delay line (Device 3), as shown schematically in Fig. 1(c). All devices presented here use monolayer CVD graphene, which is well known to exhibit inconsistent electrical characteristics between samples^28^. Subtle differences in fabrication processes can lead to different concentrations of etchant salts and PMMA residues between devices^37^, which can further affect performance. The fabrication of multiple nanoribbon arrays on the same LiNbO_3_ SAW delay line both reduces the variability in quality between different width ribbons, but also ensures that the different GNR arrays experience the same SAW intensity at any frequency. Arrays were defined in the acoustic beam path, with a width of 400 μm and a lateral spacing of 20 μm from any neighbouring arrays. All arrays shared a common electrical contact, A, in Fig. 1(c). The contact separation distances (individual nanoribbon widths) are as follows: A to B = 200 μm (200 nm); A to C = 260 μm (350 nm); A to D = 320 μm (400 nm); A to E = 380 μm (600 nm); A to F = 480 μm (500 nm); A to G = 420 μm (300 nm); A to H = 360 μm (100 nm). As in Devices 1 and 2, a perpendicular GNR bridge of 500 nm width was placed every 10 μm in each array.

Current-voltage measurements at room temperature, under vacuum, revealed that contacts A and H were open circuit, and that the array between contacts A and G had a relatively high sheet resistance of 38.8 kΩ/□. Measurements of I_ae_ from these arrays are therefore not presented. Arrays of 200, 350, 400, 500, and 600 nm-wide GNRs had sheet resistance of 15.1, 3.7, 3.8, 5.4, and 7.0 kΩ/□ respectively.

**Figure 4:**
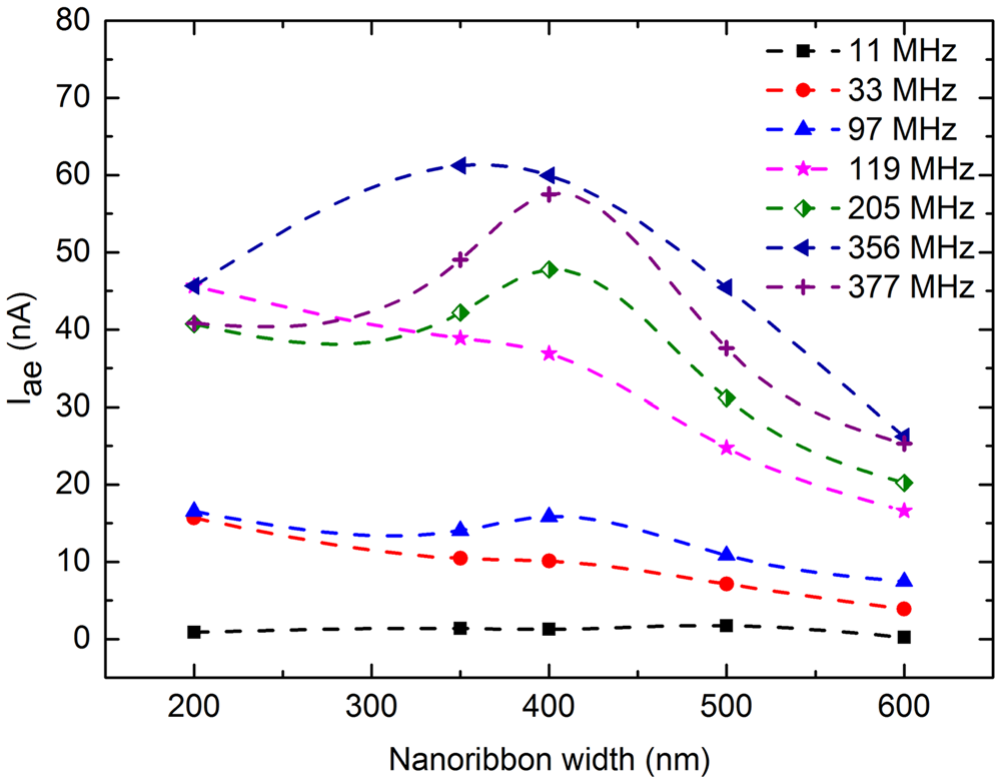
Acoustoelectric current as a function of graphene nanoribbon width, for several SAW frequencies. As GNR width increases, the acoustoelectric current at a given frequency generally decreases. Dashed lines are guides to the eye.

In Fig. 4, the acoustoelectric current is plotted as a function of GNR width and SAW frequency, at an applied RF power of +20.0 dBm, for Device 3. Dashed lines are to aid interpretation of the results. As expected from Equation (2), for a given GNR width, the acoustoelectric current increases approximately linearly as the SAW frequency increases, suggesting that the classical relaxation model is applicable to GNRs of the widths considered here. Additionally, for all GNR widths the highest acoustoelectric current is measured at *f*_SAW_ = 356 MHz, consistent with the measurements in Figs 2(b) and 3, and again believed to be due to enhanced momentum transfer from the SAWs to the GNR charge carriers when *λ*_*SAW*_ coincides with the periodicity of the perpendicular bridge structures in the arrays. This is the case even without accounting for the frequency response of the IDTs. For SAW frequencies of 11, 33, 97, and 119 MHz the acoustoelectric current generally decreases as the ribbon width increases, so that the largest acoustoelectric current is measured in the smallest ribbons. At SAW frequencies above 205 MHz I_ae_, decreases significantly when the ribbon width is decreased from 350 nm to 200 nm. A Raman map of the graphene 2D and G peaks taken at a random location in the array of 200 nm-wide GNRs revealed spatial discontinuities of ~15 μm across (see Supplementary Information). As the attenuation of the SAWs by the charge carriers is determined by the conductivity of the graphene on the scale of half the SAW wavelength^40^, and for *f*_SAW_ = 205 MHz the SAW wavelength is ~20 μm, the inhomogeneous conduction path in the 200 nm array leads to lower conductivity, lower SAW attenuation, and correspondingly lower acoustoelectric currents at high SAW frequencies, where the dimensions of the inhomogeneities are similar to the SAW wavelength. At frequencies below 205 MHz, where the SAW wavelength is much larger than the scale of the discontinuities, the acoustoelectric current increases when the ribbon width decreases from 350 nm to 200 nm, consistent with the overall trend of increasing current with decreasing ribbon width (note that results at 11 MHz are shown for completeness, but the relatively low acoustoelectric current in this case means that the changes with ribbon width are not experimentally significant).

**Figure 5:**
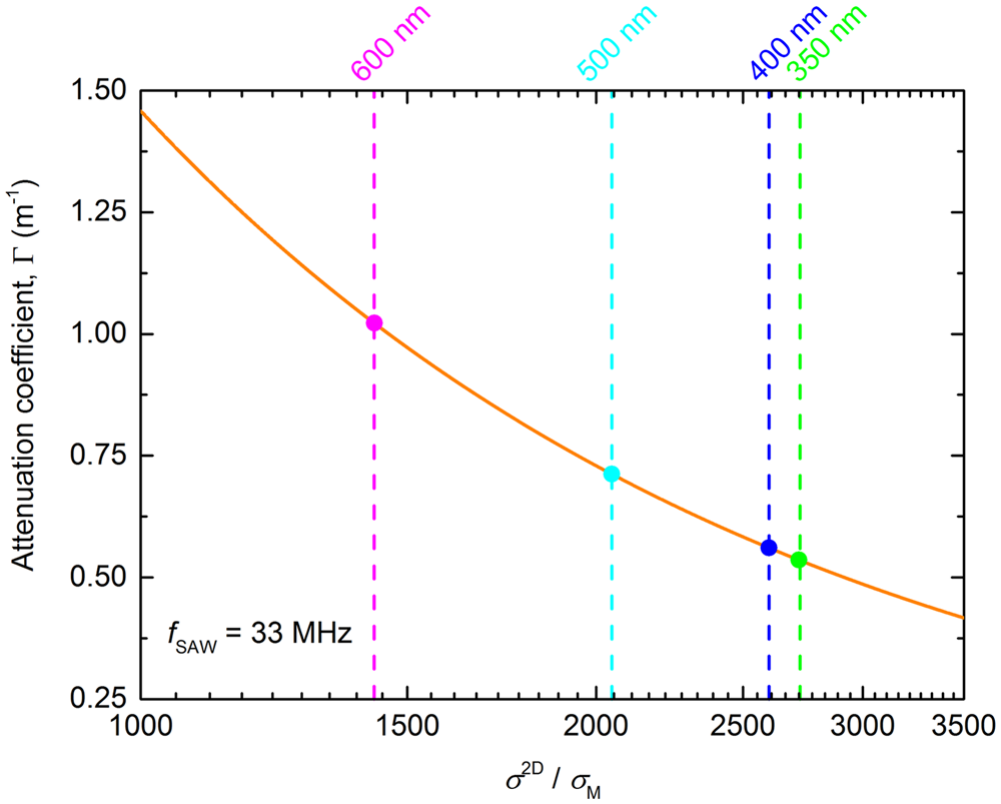
Calculated attenuation coefficient, as a function of conductivity normalised to the characteristic conductivity *σ*_M_, is shown by the solid orange line for *f*_SAW_ = 33 MHz. For each GNR array, a value of Γ has been calculated from the estimate of *σ* ^2D^ and is indicated by a coloured point. Dashed lines of the same colour indicate the conductivity of that array.

In Fig. 5, the calculated attenuation coefficient Γ (Equation 2), for *f*_SAW_ = 33 MHz, is plotted as a function of *σ* ^2D^/*σ*_M_ (solid orange line), where we have taken a value of *σ*_M_ = 10^−7^ Ω^−1^, determined for graphene-LiNbO_3_ hybrids^16^. The symbols in this plot correspond to the attenuation calculated for each GNR, again using Equation 2, where the conductivity of each GNR array (indicated by the coloured vertical dashed lines) was taken from the current-voltage measurements, assuming that each array contained no breakages and fully covered the intended area (due to the discontinuities observed in the Raman map, we have therefore not plotted the attenuation for the 200 nm ribbons). The increase in conductivity as the ribbon width decreases from 600 to 350 nm leads to the attenuation coefficient halving, in contrast to the acoustoelectric current, which increases as the ribbon width decreases. From Equation 1, this implies that as the ribbon width decreases, not only the conductivity, but also the mobility in the ribbons increases.

The increase in conductivity with decreasing GNR width (Fig. 5) is consistent with measurements made in graphene nanoconstrictions, also fabricated from monolayer CVD graphene patterned using plasma etching, of comparable widths^43^. In this study, significant p-type doping was observed as the width of the nanoconstriction was decreased, which was attributed to the oxidation of the edges of the constrictions caused by the fabrication process. The increase in the p-type doping was reflected in a shift in the Dirac voltage, with a shift of approximately 3 volts seen between 600 nm and 300 nm wide constrictions. Using the standard capacitor model for graphene on Si/SiO_2_, this shift in the Dirac point corresponds to a significant increase in the Fermi energy of approximately 15 meV. The conductivity of transferred CVD graphene is known to be limited by grain boundaries, tears, wrinkles and non-homogenous impurities^40, 42^, and in previous temperature dependent measurements of the acoustoelectric current^10^, we showed that the mobility was thermally activated, with an activation energy of approximately 60 meV. Although we are unable to directly measure the carrier concentration or Fermi energy in our measurements, an increase in the Fermi energy of 15 meV would cause the mobility to more than double (taking the activation energy to be 60 meV), when the ribbon width is reduced from 600 nm to 300 nm. This is broadly consistent with our measurements, where an increase in the mobility of this order is required to explain the observed increase in the acoustoelectric current. Lee *et al*.^44^ also showed how a non-uniform carrier concentration in the graphene can be used to explain the origin the dependence of the mobility on channel width.

Although we cannot directly measure the mobility in these devices, the magnitude of the currents observed in Device 3 (Fig. 4) are similar to those observed in samples with *μ* ~ 5–10 cm^2^/V.s^9–11^. Finally, we believe that the relatively large acoustoelectric currents measured in the array of 200 nm-wide GNRs arise from the dominant effect of poor conductivity leading to increased momentum transfer from the SAW to the graphene charge carriers, rather than the increased mobility arising from increased p-doping seen in the other arrays.

Further work is now underway to examine the width dependence of the acoustoelectric effect in graphene nanoribbons that are electrostatically gated^16^, and better understand the role of GNR edges on the mobility and conductivity of these systems. The charge carrier conduction mechanism in graphene arises from several competing mechanisms, and the role of the LiNbO_3_ substrate in this process is not yet fully understood. We also intend to investigate the effects of changing the bridge periodicity on the acoustoelectric current, tailoring it to coincide with other SAW wavelengths, for example.

## Conclusion

In conclusion, we have demonstrated that the acoustoelectric effect in monolayer CVD graphene nanoribbons on LiNbO_3_ SAW delay lines, with widths up to 600 nm, is consistent with the description of this interaction by a relatively simple classical relaxation model. In 300 μm × 3 mm arrays composed of 500 nm-wide GNRs, an acoustoelectric current of up to ~5.5 μA was measured at a SAW frequency of 442 MHz. Enhanced acoustoelectric currents were observed when perpendicular bridge structures were inserted into the nanoribbon array with a period similar to the SAW wavelength. The increasing acoustoelectric current with decreasing nanoribbon width is thought to be caused by increased doping along the damaged, rough edges introduced by the fabrication process. We believe these measurements provide a stepping stone towards the eventual use of the acoustoelectric effect in graphene nanoribbons for such applications as sensing and metrology.

## Methodology

The devices measured here were fabricated as follows: Via the poly(methyl methacrylate) (PMMA) transfer technique^45^, monolayer graphene produced by chemical vapour deposition on ~5 mm × ~5 mm copper foils (Graphene Supermarket) was transferred in-between the interdigital transducers (IDTs) of commercially available 128° YX LiNbO3 SAW delay lines. These had identical input and output aluminium IDTs defining an acoustic beam width of 3.25 mm and a path length of 5.4 mm. Patterning of the graphene was undertaken using electron beam lithography and an argon (10 W) reactive ion etch. GNRs were aligned such that their long axes lay parallel to the SAW wave vector. For all devices, 20 μm-wide Cr/Au (5 nm and 50 nm respectively) electrical contacts were deposited by thermal evaporation.

Devices supporting 500 nm-wide GNRs only (schematically shown in Fig. 1(a)) were patterned into 2 mm × 3 mm nanoribbon arrays positioned centrally between the IDTs; contacts A and B, B and C, and C and D are separated by 300 μm, 200 μm, and 500 μm respectively. Devices consisting of GNR arrays made up of ribbons of different widths (schematically shown in Fig. 1(c)) were patterned in the acoustic path next to each other as described in the Results section.

The presence of monolayer graphene on the substrate was confirmed using a confocal Raman microscope (WITec Alpha300) equipped with a thermoelectrically cooled CCD detector. A 532 nm wavelength laser was used for the excitation, and a 50x objective lens was used for backscattered light collection with a lateral resolution of 388 nm. The laser power incident on the sample was approximately 2.3 mW, and the 2D and G peaks were located at 2696 cm^−1^ and 1590 cm^−1^ respectively, similar to values reported by Gupta *et al*.^38^ (Raman maps of each peak for an array of 500 nm-wide GNRs (Device 1) are shown in the Supplementary Information).

All measurements were made under vacuum (chamber pressure approximately 6 × 10^−6^ mbar) with continuous pumping to reduce the accumulation of dopants, at room temperature. Continuous wave SAWs were excited at the input transducer using a Hewlett-Packard 8648C RF signal generator, while Iae was measured using a Keithley K2400 source-measurement unit. Two-terminal current-voltage measurements were used to determine the GNR array conductivities. GNR arrays that were not being characterised were electrically grounded to reduce interference. The SAW amplitude at the output IDT was measured using a LeCroy WaveRunner 204Xi-A digital oscilloscope, and used to estimate SAW intensity.

## Electronic supplementary material

Supplementary materials
