# Supplementary material for: Acoustoelectric Current in Graphene Nanoribbons
Source: Sci Rep. 2017 May 11;7:1767. doi: 10.1038/s41598-017-01979-8 (PMC5431792; doi:10.1038/s41598-017-01979-8)
Supplement: Supplementary file 1 — Supplementary materials [file 41598_2017_1979_MOESM1_ESM.pdf]

Acoustoelectric Current in Graphene Nanoribbons
Supplementary Information

T. Poole and G. R. Nash*

g.r.nash@exeter.ac.uk

The relative amplitude of surface acoustic waves is plotted as a function of SAW frequency in Figure S1. The voltage across the output IDT of the SAW devices was measured with a LeCroy WaveRunner 204Xi-A digital oscilloscope and compared to the input voltage, supplied by the Hewlett-Packard 8648C signal generator. The orange curve shows measurements made on Device 1 which had 500 nm-wide bridge structures inserted in the GNR array every 10 μm. At SAW frequencies with a wavelength commensurate with the periodicity of the bridge structures (356 – 377 MHz, SAW wavelength ~11.2 – 10.6 μm) there is a sharp decrease of approximately 15 dB in relative amplitude (to a relative amplitude of -75 dB). The relative amplitude increases slightly again at higher frequencies to around -70 dB. The green curve shows identical measurements performed on a similar device that did not include the bridge structures. In this case, there is no such decrease in relative SAW amplitude. This, we believe, indicates the role of the bridge structures in enhancing the piezoelectric interaction between the graphene charge carriers and the SAWs.

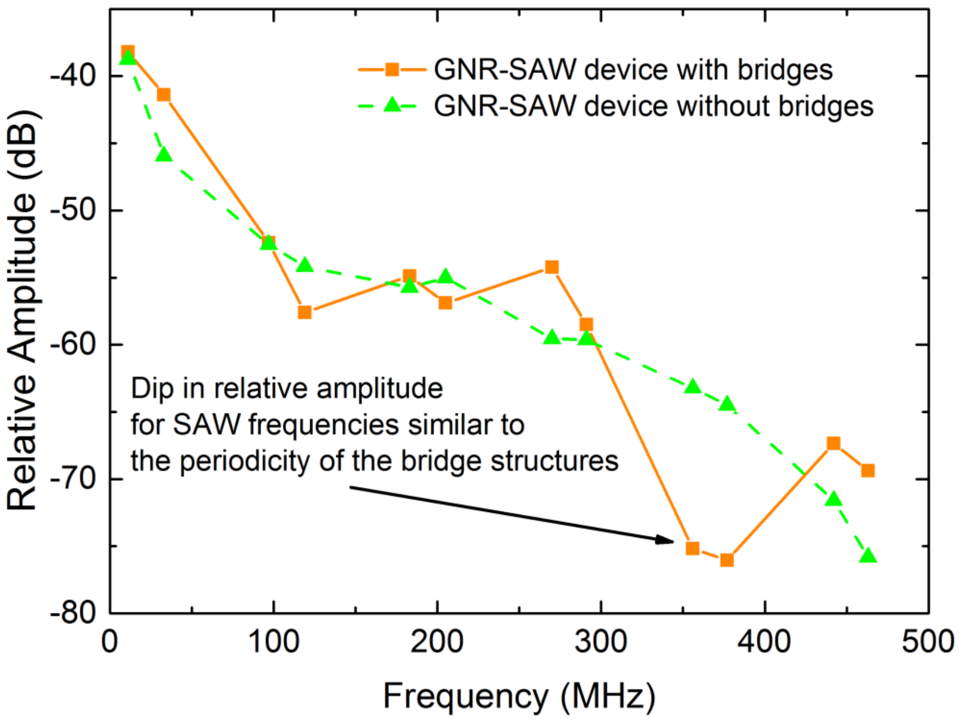

Figure S1: Relative SAW amplitude as a function of SAW frequency. Orange, square points correspond to measurements of a GNR-SAW device in which 500 nm-wide bridge structures had been inserted into the GNR array every 10 μm. There is a dip in relative amplitude when the SAW wavelength is commensurate with the bridge periodicity (SAW frequencies 356-377 MHz). Green, triangular points show identical measurements made in a GNR-SAW device without the bridge structures. No dip in relative SAW amplitude is seen.

In Figure S2, the acoustoelectric current is plotted as a function of SAW frequency for the same SAW intensity as in Figure 3, in a GNR-SAW hybrid device with a 3 mm x 2 mm array of 500 nm-wide GNRs that do not include the bridge structures. For a SAW frequency of 356 MHz there is no sharp increase in acoustoelectric current, in contrast to the measurements in Figure 3 carried out on a bridged device. This is reflected the relative SAW amplitude measurements for this device in Figure S1 (green curve), where the relative SAW amplitude does not exhibit a large decrease at these SAW frequencies. This further supports the idea that

the bridge structures enhance the piezoelectric interaction between the graphene charge carriers and the SAW, leading to the generation of larger acoustoelectric currents.

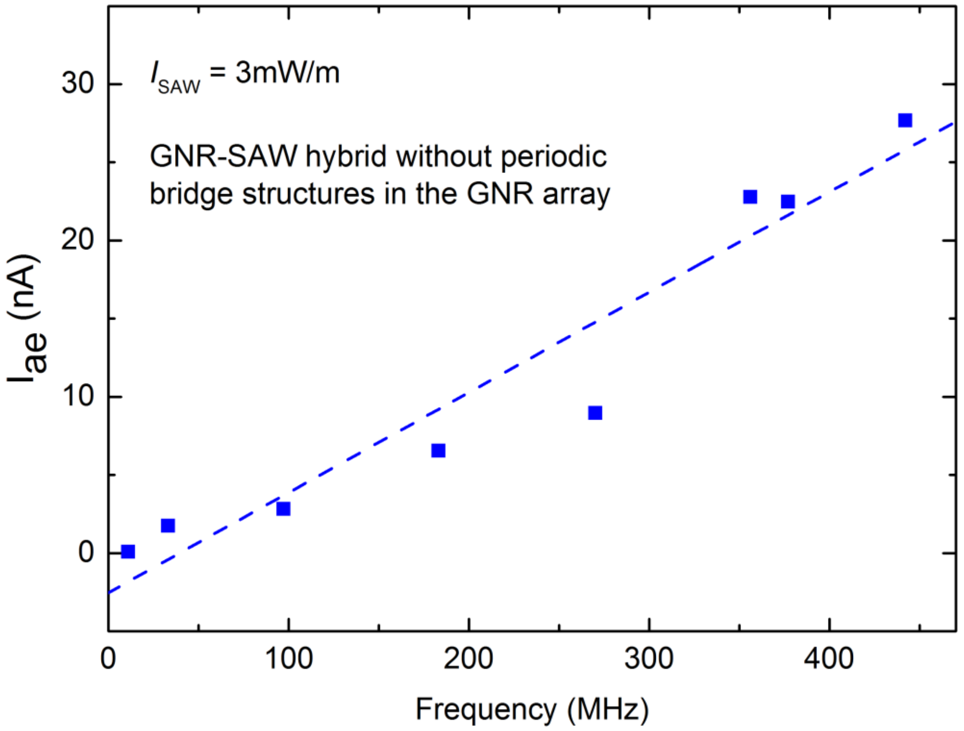

Figure S2: Acoustoelectric current as a function of SAW frequency for a GNR-SAW device that does not include periodic bridge structures in the GNR array. GNRs are of 500 nm width and is identical to Device 1 in all other respects. The smaller measured acoustoelectric current reflects the relatively high resistance (0.91 MΩ) of the graphene nanoribbon array on this device.

The presence of monolayer graphene on the substrates was confirmed using a confocal Raman microscope (WITec Alpha300) equipped with a thermoelectrically cooled CCD detector. A 532 nm wavelength laser was used for the excitation, and a 50x objective lens was used for backscattered light collection with a lateral resolution of 388 nm. The laser power incident on the sample was approximately 2.3 mW. Figure S3 shows an optical image of Device 1. The two white stripes are electrodes A and B in Figure 1, separated by 300 μm and each having a width of 20 μm. In contrast to graphene on Si/SiO_2, optical microscopy does not reveal the location of graphene sheets on $LiNbO_3$ and Raman spectroscopy is needed to survey the graphene quality. The green rectangle indicates some damaged graphene, visible as black marks as it has torn and rolled into multi-layers. The red square indicates the randomly selected region for which a Raman spectrum was taken.

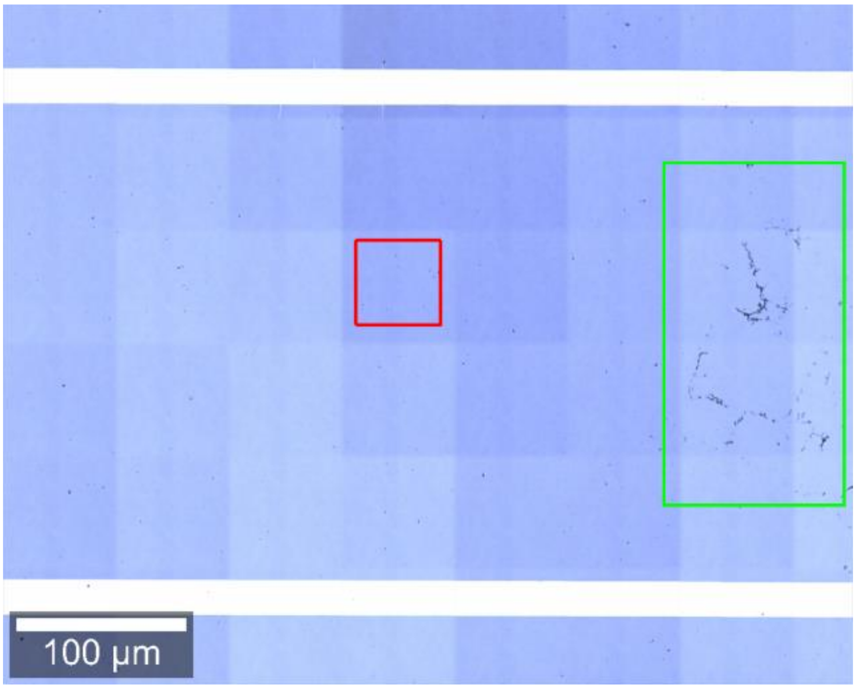

Figure S3: Optical image of Device 1 between contacts A and B in Figure 1. The chequered appearance arises from stitching multiple images together.

Figure S4 shows an average Raman spectrum of 10 randomly chosen points corresponding to positions of graphene nanoribbons in Device 1 between contacts A and B, selected from a colour plot of the 2D peak. Background subtraction and cosmic ray removal was performed to aid interpretation of the results. The positions of the 2D and G peaks are consistent with reports by Gupta *et al.* [S1], and the blue shift of the 2D-peak and G-peak is consistent with the p-doping of graphene nanoconstrictions [S2].

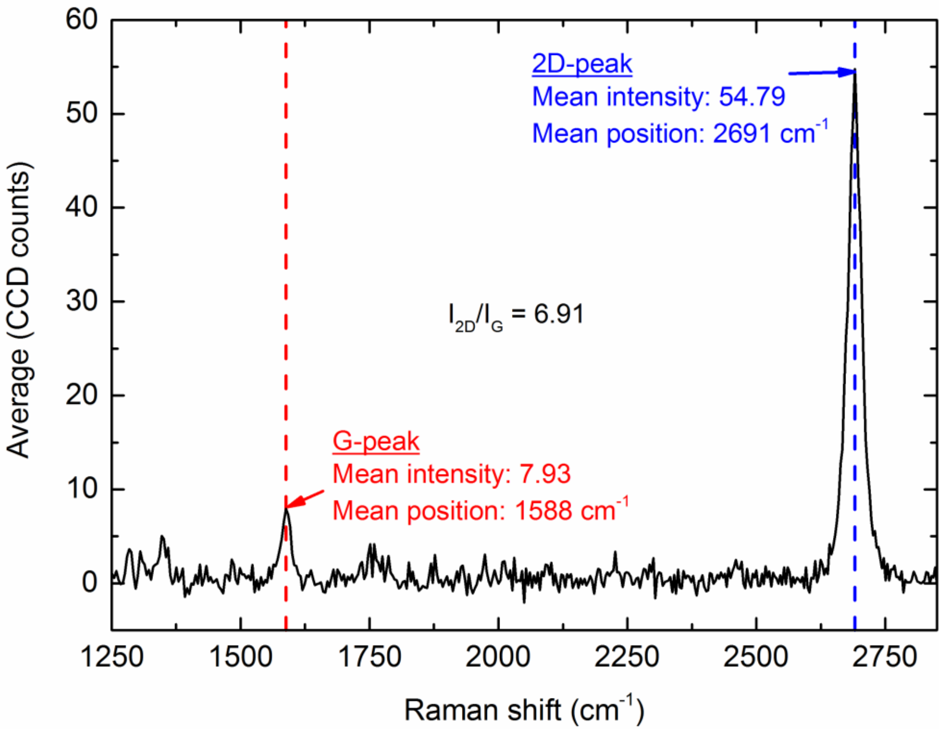

Figure S4: Raman spectrum of 10 randomly chosen points in the GNR array in Device 1, between contacts B and C.

Two-dimensional spatial maps of the 2D and G peaks are plotted in Figure S5(a) and S5(b) respectively. The GNR array structure is clearly visible, and there are some key features that have been highlighted in Figure S5(a). The horizontal lines spaced every 10 μm (indicated by pink squares) correspond to the perpendicular bridge structures included in the array to enhance electrical conductivity. The increased intensity of the 2D and G peaks in these regions is likely due to under-dosing in these regions. Bright vertical lines (indicated by green circles) arise due

to slight stitching errors during the electron beam lithography; the spacing of 20 μm corresponds to the sub-field stitching size of our lithography system. The blue rectangle indicates a piece of graphene that has rolled into a multilayer structure; a two-dimensional plot of the D peak shows that there is significant disorder associated with this feature. Elsewhere, disconnected GNRs are visible via dark patches in each image. A wrinkle can also be seen running vertically in both Figures (again, this feature is associated with a high intensity D peak compared with the surrounding graphene).

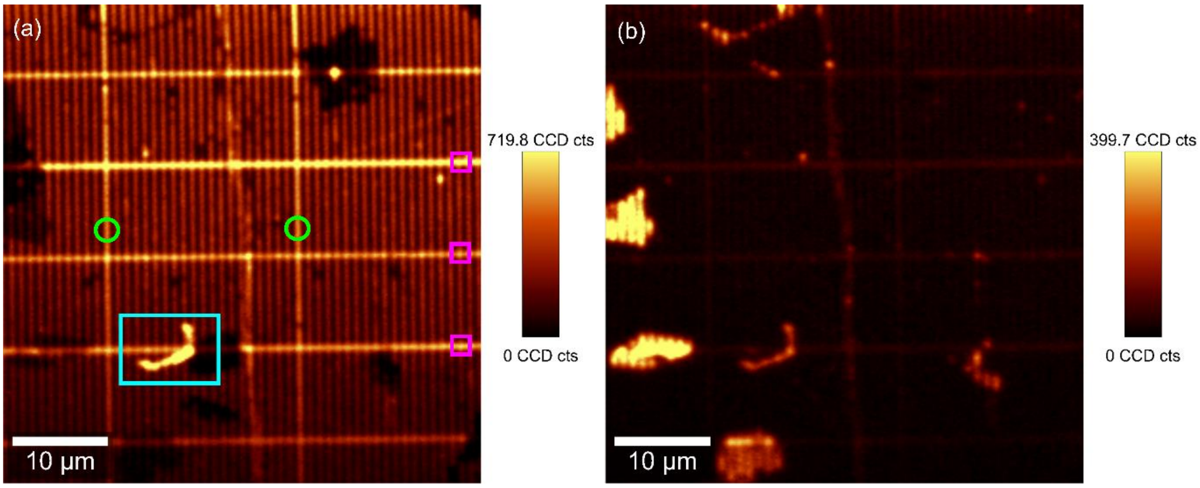

Figure S5: In (a) and (b) the 2D and G peaks respectively are plotted for Device 1, for the region indicated by the red square in Figure S1.

Figure S6 shows an optical image of Device 3 between contacts A and B (horizontal white lines), and the red square indicated the randomly selected region in which for which Raman spectra were taken. The white line at the top of the image is contact C. The small flake of metal above the red square is from incomplete lift-off of the Cr/Au thermally deposited when defining the electrodes.

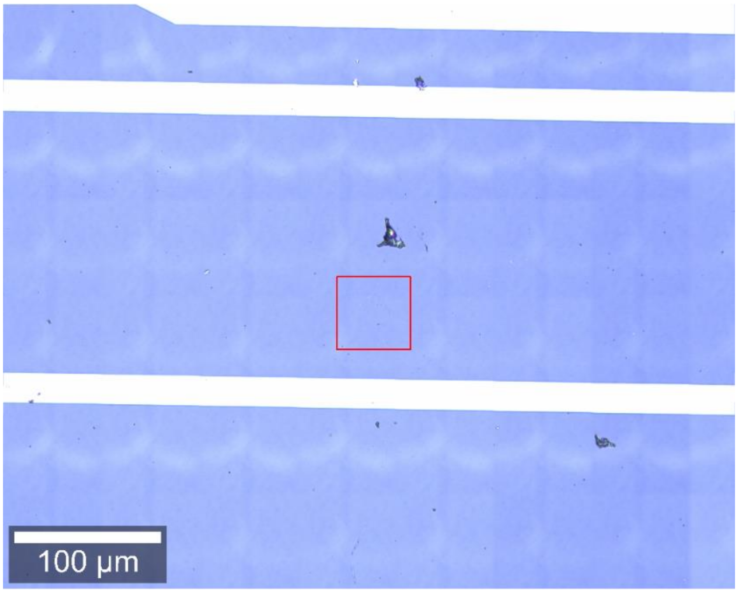

Figure S6: Optical image of Device 3 between contacts A and B. The red square indicates the region in which a Raman spectrum was taken.

The poor conductivity of the array of 200 nm-wide GNRs in Device 3, and the low acoustoelectric current measured in this array for SAW frequencies of 205, 356, and 377 MHz, was thought to be caused by an inhomogenous conduction pathway. Graphene on $LiNbO_3$ is not visible under optical microscopy, which presents difficulty in confirming this hypothesis. Raman analysis is a convenient tool to see the scale of any damage in the graphene. In Figure S7(a) and (b), two-dimensional maps of the 2D (2689 cm^{-1}) and G (1590 cm^{-1}) peaks are plotted. As in Figure S5(a) and (b), horizontal lines correspond to the bridge structures designed to help maintain electrical continuity in the array. Due to the size of the probe laser (388 nm), individual GNRs are not resolvable. This results in broader 2D and G peaks in the Raman spectra. More importantly, however, there is significant spatial inhomogeneity indicated by the blue rectangle in Figure S7(a), as well as other cracks, wrinkles and scratches. This evidence supports the theory that the relatively small acoustoelectric current observed at SAW frequencies above 205 MHz in this array arise from the SAW probing the conductivity of the graphene on different length scales.

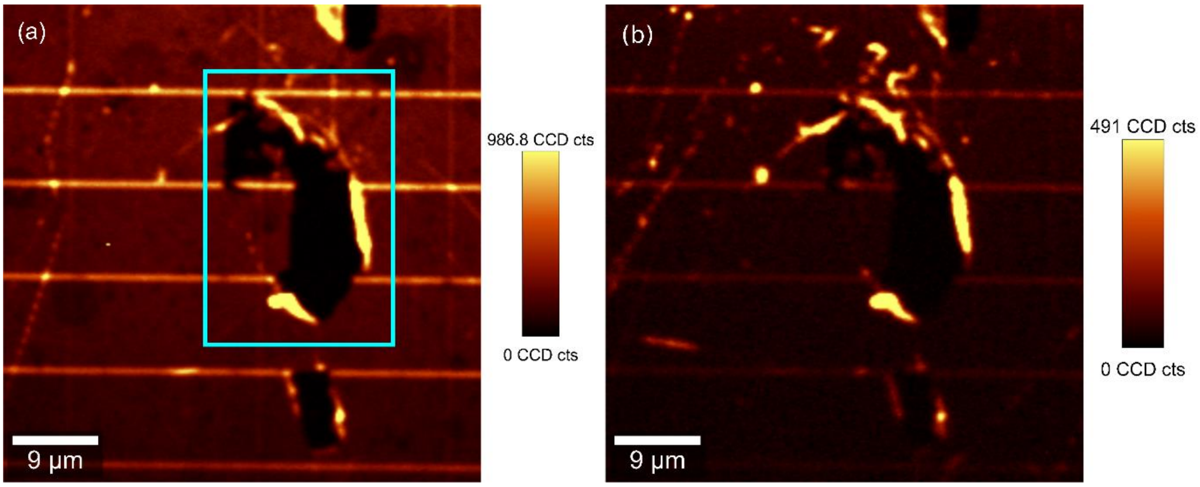

Figure S7: In (a) and (b) the 2D and G peaks respectively are plotted for Device 3, for the region indicated by the red square in Figure S4.

References

[S1] Gupta, A., Chen, G., Joshi, P., Tadigadapa, S. & Eklund, P. C. Raman scattering from high-frequency phonons in supported n-graphene layer films. *Nano Lett.* **6,** 2667 - 2673 (2006).

[S2] Iqbal, M. W., Iqbal, M. Z., Jin, X., Hwang, C. & Eom, J. Edge oxidation effect of chemical-vapor-deposition-grown graphene nanoconstriction. *ACS Appl. Mater. Interfaces* **6,** 4207 – 4213 (2014).
